# Targeting HSP47 and HSP70: promising therapeutic approaches in liver fibrosis management

**DOI:** 10.1186/s12967-022-03759-z

**Published:** 2022-11-26

**Authors:** Eslam E. Abd El-Fattah, Amr Y. Zakaria

**Affiliations:** 1 Department of Biochemistry, Faculty of Pharmacy, Delta University for Science and Technology, Gamasa, Egypt; 2 Clinical Pharmacy (Pharmacy Practice) Department, Faculty of Pharmacy, Horus University, New Damietta, Egypt

**Keywords:** Liver fibrosis, Collagen, HSP70, And HSP47

## Abstract

Liver fibrosis is a liver disease in which there is an excessive buildup of extracellular matrix proteins, including collagen. By regulating cytokine production and the inflammatory response, heat shock proteins (HSPs) contribute significantly to a wider spectrum of fibrotic illnesses, such as lung, liver, and idiopathic pulmonary fibrosis by aiding in the folding and assembly of freshly synthesized proteins, HSPs serve as chaperones. HSP70 is one of the key HSPs in avoiding protein aggregation which induces its action by sending unfolded and/or misfolded proteins to the ubiquitin–proteasome degradation pathway and antagonizing influence on epithelial-mesenchymal transition. HSP47, on the other hand, is crucial for boosting collagen synthesis, and deposition, and fostering the emergence of fibrotic disorders. The current review aims to provide light on how HSP70 and HSP47 affect hepatic fibrogenesis. Additionally, our review looks into new therapeutic approaches that target HSP70 and HSP47 and could potentially be used as drug candidates to treat liver fibrosis, especially in cases of comorbidities.

## Introduction

Liver fibrosis is an inflammatory response brought on by a variety of conditions, including alcohol use, non-alcoholic steatohepatitis (NASH), viral hepatitis (hepatitis B (HBV) and hepatitis C (HCV)), autoimmune hepatitis, non-alcoholic fatty liver disease (NAFLD), and cholestatic liver diseases [1]. The formation of a chronic inflammatory response results in an aberrant wound healing response which induces extracellular matrix (ECM) components accumulation in the liver and thus the creation of fibrous scar tissue. The existence of a fibrous scar alters the architecture of the liver, leading to hepatocyte loss, the disruption of normal liver function, and ultimately liver failure [2, 3]. Unless it progresses and becomes cirrhosis, liver fibrosis can be reversed.

Liver diseases whatever result from pathogenic, toxic, metabolic, or viral causes induce hepatocyte damage and immune cell infiltration that activates the trans-differentiation of hepatic stellate cells (HSCs) into myofibroblasts that produce collagen [4]. HSCs differentiate into myofibroblasts, begin expressing alpha-smooth muscle actin (α-SMA), move to tissue healing sites, and secrete a considerable amount of ECM [5]. Myofibroblasts may undergo apoptosis and inactivation after the liver injury is removed [6].

Through a process known as epithelial-mesenchymal transition (EMT), epithelial cells that are normally found on the exterior of blood vessels and organs may lose their polarity, migrate, and give rise to myofibroblasts [6]. Interestingly, Xie and Diehl [7] found that extended culture of cholangiocytes and hepatocytes increases the expression of α-SMA while decreasing the expression of epithelial markers.

Transforming growth factor-beta 1 (TGF-β_1_) mediates the activation of portal fibrosis during cholestatic liver fibrosis, which includes the interaction of mesothelin with a MUC16-Thy1- TGF-β_1_RI complex [8, 9] and platelet-derived growth factor (PDGF), as well as increased contractility, and high levels of α-SMA, and connective tissue growth factor (CTGF) [10].

Myofibroblasts are physiologically implicated in tissue regeneration; however, after a short-term insult, anti-fibrotic mechanisms balance this activity, leading to myofibroblast inactivation or apoptosis and scar resolution. In contrast, chronic liver disorders result in prolonged activation of proliferative, contractile, and migratory myofibroblasts that result in an excess synthesis of ECM due to an imbalance of pro- and anti-fibrogenic pathways. ECM consists of type I and III collagen and fibronectin and its presence depends on the balance between matrix metalloproteinase (MMP) and tissue inhibitors of metalloproteinases (TIMP). ECM predominates as MMPs activity decreases and TIMPs activity increases [11].

AMPK (5' adenosine monophosphate-activated protein kinase) is a metabolic master regulator that regulates cellular energy homeostasis [12]. AMPK activity has been shown to suppress HSC activation by decreasing the activation of either nuclear factor kappa B (NF-kB) or mammalian target of rapamycin (mTOR) signaling [13]. In mice, adiponectin (an AMPK activator) deficiency exacerbated CCl4-induced fibrosis [14]. Furthermore, activation of AMPK in human HSCs by adiponectin or 5-Aminoimidazole-4-carboxamide ribonucleoside (AICAR) reduced HSC activation and migration in response to PDGF [13]. Likewise, inhibiting AMPK boosted PDGF-induced HSC proliferation, migration, and activation [13].

Non-parenchymal cells (NPCs), such as Kupffer cells and other immune cells, play a major role in determining whether the liver enters an anti-fibrotic scar-dissolving stage or advances into an unchecked fibrosis-promoting stage. Hepatocyte apoptosis and the release of damage-associated patterns (DAMPs) also cause the recruitment and activation of lymphocytes and macrophages, which promote HSC trans-differentiation and myofibroblast activation by producing pro-inflammatory and pro-fibrogenic cytokines to induce inflammation, such as PDGF, tumor necrosis factor-alpha (TNF-α), and interleukin-1 beta (IL-1β), as well as activating the TGF-β_1_/Smad signal pathway, mitogen-activated protein kinase (MAPK) [15–18].

Additionally, by producing and secreting pro-inflammatory and pro-fibrogenic chemicals including apoptosis-signal-regulating kinase 1, pan-caspase, and galectin-3, monocytes further damage hepatocytes, encourage the activation of HSCs, and exacerbate inflammation and fibrosis. TGF-β_1_ also promotes the conversion of monocytes into macrophages which secrete inflammatory mediators like IL-1 and IL-6 that encourages the escalation of the inflammatory response and the ongoing activation and survival of HSCs [19].

Besides all these HSCs activators, there is a promising approach that activates HSCs through control of different heat shock proteins (HSPs) levels and is thus considered a new therapeutic target for the management of liver fibrosis.

### HSPs and liver fibrosis

Heat shock proteins are stress proteins that cannot be activated under normal conditions because their expression is rigorously controlled by a variety of environmental and physiological insults, such as heat shock, oxidative stress, heavy metals, ultraviolet radiation, and membrane perturbations, either to aid in cell survival or to promote the death of an irreparably damaged cell [20, 21]. Based on their molecular weights, the HSPs family is divided into HSP100, HSP90, HSP70, HSP60, and HSP47. In fibrosis, HSPs are crucial for collagen formation in addition to their roles in anti-oxidation, synergistic immunity, and anti-apoptosis. HSPs may therefore be intimately linked to the development or the prevention of fibrogenesis and fibrosis [22]. Among the most prevalent HSPs that affect liver fibrogenesis are the pro-fibrotic HSP47 and the anti-fibrotic HSP70.

#### A-Role of HSP70 in the regulation of liver fibrosis

In response to numerous stimuli, such as heat, oxidative stress, and chemical damage, HSP70 is up-regulated in cells to help in avoiding protein aggregation [23]. Additionally, EMT, a player in the fibrosis process, is negatively impacted by HSP70 [24]. Sellares and Veraldi [25] mentioned that Hsp70 deficiency contributes to fibrosis, and interventions aimed at restoring normal Hsp70 expression represent a novel therapeutic strategy for fibrosis.

#### B-Role of HSP47 in the regulation of liver fibrosis

HSP47 is presented in the endoplasmic reticulum and is crucial in controlling collagen synthesis. HSP47 is implicated in fibrotic disorders such as scleroderma, renal interstitial fibrosis, peritoneal fibrosis, cardiac fibrosis, intestinal fibrosis, keloid fibrosis, and pulmonary fibrosis by encouraging the buildup of collagen [26].

Chronic hepatitis B and chronic schistosomiasis patients both had elevated levels of HSP47, TGF-β1, and CTGF. HSP47 mRNA expression considerably increased as schistosomiasis hepatic fibrosis progressed [27, 28] which makes HSP47 a biomarker for schistosomal hepatic fibrosis in its early stages [29, 30].

Additionally, HSP47-targeted small interfering RNA (siRNA) and short hairpin RNA (shRNA) can reduce collagen formation in mice with hepatic fibrosis brought on by Schistosoma japonicum [31].

The expression of HSP47, endothelin receptor A (ETAR), and endothelin receptor B (ETBR) was significantly increased in mice models of liver fibrosis caused by Schistosoma japonicum. When HSP47 shRNA was applied in vitro and in vivo, HSP47 expression was significantly reduced which decreased ETAR and ETBR levels on the cell membrane surface [32].

HSCs are the source of HSP47 expression and blocking HSCs activation can diminish the synthesis of HSP47, hence limiting or avoiding liver fibrosis [32]. HSP47 inhibition significantly suppressed collagen production in fibroblasts in vitro in the ulcerative colitis model [33]. HSP47 synthesis may be regulated by heat shock factor 1 (HSF-1) activation. Inactivation of HSF1 by both tumor necrosis factor (TNF)-related apoptosis-inducing ligand (TRAIL) and siRNA results in the down-regulation of HSP47, lowering collagen buildup, and delaying the fibrosis process [34].

Here, we will discuss different regulators of Both HSP70 and HSP47 that aids in controlling liver fibrosis.

### Factors regulate HSPs activity

#### TGF-β/Smad4 signaling pathway and HSPs

MiR-455-3p decreases HSF-1 expression and limits HSC activation by inhibiting the HSP47/TGF-β_1_/Smad4 signaling pathway [35]. Furthermore, miR-125b, miR-378, and miR-152 can prevent liver fibrosis by modulating GLI family zinc expression [36].

TGF-βIR can phosphorylate SMAD2 and SMAD3, which suggests that phosphorylation of SMAD2 and SMAD3, is required for the smooth transmission of the TGF-β signaling pathway and thus its activation and accelerating the development of liver fibrosis [37, 38]. Hsp70 can inhibit the phosphorylation of these two SMADs by interacting with SMAD2 and SMAD3, thereby inhibiting the conduction of the TGF-β signaling pathway [39].

#### Nuclear receptors (NRs) and HSPs

The removal of a stabilizing HSP aimed directly at gene transcription in the cell nucleus activates type-1 NRs before dimerization in the cytoplasm [40]. Hsp40-induced ATP hydrolysis delivers protein substrate to Hsp70 and increases Hsp70 association with a cochaperone, Hsc-70-interacting protein (Hip). BCL-2-associated athanogene-1 (BAG-1) is another cochaperone protein that can attach to Hsp70, displacing Hip from the heat shock complex [41, 42]. BAG-1 and similar polypeptides are ubiquitin-like proteins that can directly connect HSP70 and its client to the 26S proteasome. It has been postulated that BAG-1 may transport an hsp70 client close to the proteasome and guide substrates to the 26S proteasome for decomposition [43].

On the other hand, HSP47 is accumulated at many binding sites along the triple helix rather than being released at the end of procollagen folding. It could also act as an adaptor for TANGO1's procollagen loading into endoplasmic reticulum exit sites (ERESs) [44]. As a result, it is widely assumed that HSP47 is co-transported with procollagen from the ER to the ERGIC or cis-Golgi, where it is released at lower pH [45, 46].

#### Chaperone-mediated autophagy (CMA) and HSPs

Chaperone-mediated autophagy (CMA) is one of the primary proteolytic pathways of the lysosome-autophagy system. CMA is a type of selective autophagy in which the proteins targeted for breakdown must have a unique pentapeptide pattern recognized by HSC70/HSPA8 [47]. The chaperone-bound proteins are then delivered to lysosomes, where the lysosome-associated membrane protein type 2a (LAMP2a) receptor recognizes them [47]. CMA transports proteins for lysosomal breakdown one at a time. In contrast, autophagosomes engulf and deliver bigger structures for bulk cargo breakdown in macro-autophagy [47].

#### HSC and HSPs

HSCs can return to an inactive/quiescent state during liver fibrosis regression [6]. Approximately 50% of hepatic myofibroblasts escape apoptosis and revert to a quiescent-like phenotype during fibrosis recovery, downregulating fibrogenic genes and upregulating the survival proteins Hspa1a/b [6]. In fibrotic mice, transcriptional reprogramming by ectopic expression of the transcription factors FOXA3, GATA4, HNF1A, and HNF4A causes mouse myofibroblasts to transdifferentiate into hepatocyte-like cells resulting in reduced liver fibrosis [48].

#### ECM Remodeling and HSPs

ECM remodeling targeting is an effective method. MMP-mediated and macrophage-mediated ECM breakdown may be beneficial. Feng, Ding [49] revealed that in a mouse model of liver fibrosis, Kupfer cells (KCs) depletion delayed resolution, and adoptive transfer of KCs from WT animals expedited resolution compared to KCs from MMP9/ mice, implying that KC-derived MMP9 is required for fibrosis reversal. By interfering with collagen and elastin cross-linking, selective lysyl oxidase-like 2 (LOXL2) inhibitors diminish ECM stability and resistance to MMP destruction [50]. However, targeting LOXL2 in therapeutic studies with humanized anti-LOXL2 antibodies has so far yielded little clinical benefit [51]. Hsp47, a Col1 chaperone, was inhibited in liver fibrosis models by Hsp47 siRNA encapsulated in vitamin A-coupled liposomes, which are preferentially taken up by HSCs, showing anti-fibrotic effects [52]. COL1A1 and HSPs.

In addition to direct regulation of the COL1A1 gene, other proteins associated with collagen expressions, such as α-complex protein 2 (αCP2), transport and Golgi organization 1 (TANGO1), and HSP47, have been studied to treat liver fibrosis [53, 54]. The aberrant ECM buildup during liver fibrosis is associated with an increase in the half-life of the COL1A1 mRNA from 1.5 h in quiescent HSCs to more than 24 h in active HSCs.

### Positive regulators of HSP70

#### Curcumin

Curcumin promotes HSP70 expression in intestinal Caco-2 cells via various signaling pathways in intestinal epithelial cells [55]. In primary rat cortical neuronal apoptosis induced by gp120 V3 loop peptide, curcumin increases HSP70 expression [56]. Hernández-Aquino, Quezada-Ramírez found that curcumin's antifibrotic actions were produced by a decrease in activated HSCs cells as a result of normalizing the GSH, NF-kB, JNK-Smad3, and TGF-β_1_—Smad3 pathways. Saadati, Hatami [57] found that only the curcumin group experienced significant reductions in hepatic fibrosis, serum cholesterol, glucose, and glutamic-pyruvic transaminase (ALT).

#### Caffeine

In Caenorhabditis elegans, coffee extract improves HS-induced HSP-70 promoter activity [58]. Using meta-analysis, Liu, Wang [59] found that consuming coffee can greatly lower your risk of developing cirrhosis and hepatic fibrosis. Modi, Feld [60] found that in all patients, including the subset with HCV infection, daily caffeine consumption above the 75(th) percentile for the cohort (308 mg) was linked to lessened liver fibrosis.

#### Metformin

Metformin is an antidiabetic medication used to treat and prevent the polycystic ovarian syndrome, type 2 diabetes mellitus, gestational diabetes, weight gain brought on by antipsychotics, and gestational diabetes [61]. Metformin increased the expression of numerous genes, including HSP 70 in two human esophageal squamous-cell carcinoma cell lines [62]. In Lee, Lee [63] clinical trial, a cohort of patients with metformin treatment showed a small proportion of patients developed liver fibrosis and steatosis after 2 years.

#### Testosterone

The primary male hormone responsible for regulating sex differentiation, producing male sex characteristics, spermatogenesis, and fertility is testosterone. In males with cirrhosis, low testosterone is a novel prognostic sign that is statistically linked to higher mortality, the requirement for transplantation, as well as risk for serious infection. [64]. The expression of HSP70-2a, HSP90, and PCNA is increased by testosterone in the experimental varicocele condition [65]. Yassin, Alwani [66] found that long-term testosterone therapy reduces hepatic steatosis and enhances liver function in hypogonadal males.

#### Melatonin

Melatonin is produced by the pineal gland during the night in reaction to darkness. In rats, oxidative stress is thought to contribute to functional and histopathologic changes linked to chronic cerebral hypoperfusion. Melatonin has been shown to protect against cerebral ischemia or reperfusion injury. This impact has been attributed mostly to its antioxidant characteristics which are accompanied by a rise in malondialdehyde concentration and HSP70 induction [67]. Jie, Hong [68] discovered that melatonin may reduce liver fibrosis by controlling autophagy, indicating that it may be used as a treatment for liver fibrosis. Melatonin has been shown to have antifibrotic effects on the liver, reducing profibrogenic indicators and altering some cellular functions and molecular pathways. It also acts primarily as an antioxidant and anti-inflammatory agent. [69]. Tahan, Akin [70] found that bile-duct ligation caused levels of collagen, MDA, luminal, and lucigenin to rise while GSH levels fell; however, melatonin had the opposite effect.

#### N-acetylcysteine (NAC)

N-acetylcysteine treatment significantly reduced hepatic inflammation and collagen deposition, decreased serum ALT, aspartate transaminase (AST), and total bilirubin, decreased hepatic hydroxyproline and malondialdehyde (MDA), down-regulated HSP47 protein expression while increasing albumin content, and significantly improved superoxide dismutase activity (SOD) [71]. Hsp70 levels increased in MG132-treated cells when NAC was added [72]. Pereira-Filho, Ferreira [73] found that through histological investigation and collagen quantification, the cirrhotic group treated with NAC demonstrated decreased degrees of fibrosis. When compared to the cirrhotic group without therapy, this group has also demonstrated less cellular membrane deterioration, less of a drop in glutathione peroxidase levels, and less expression of inducible nitric oxide synthase.

#### Verapamil plus bortezomib

Bortezomib strongly induced Hsp70 expression, which was enhanced when combined with verapamil in myeloma cells [74]. HSP90B1 (GRP94), HSP70, ATF6, and DDIT3 were all upregulated after verapamil and bortezomib treatment in mantle cell lymphoma [75]. In comparison to the liver fibrosis model control, verapamil caused a dose-dependent decrease in blood ALT, liver malondialdehyde, and hydroxyproline. Verapamil slowed the development of liver fibrosis and decreased hepatocyte necrosis and degeneration. Three of the verapamil-treated groups had considerably lower levels of α-SMA and TGF-β_1_ expression in the hepatic tissue than the liver fibrosis model control group [76]. Bortezomib is a good drug repositioning candidate since it directly decreases renal fibrosis in CKD by suppressing TGF-β_1_-Smad3 signaling [77].

#### Geranylgeranylacetone (GGA)

Geranylgeranylacetone (GGA) is an HSP70 inducer that has been used clinically as an anti-ulcer medication for many years. In the experimental traumatic brain injury mice model, GGA increased the number of HSP70^+^ cells [78]. In CCl4-induced liver fibrosis, GGA acted favorably by increasing the expression of HSP70. In comparison to the control group, GGA prevented liver fibrosis, reduced the amount of hydroxyproline, restored liver function, downregulated the expression of pro-fibrogenic proteins α-SMA and TGF-β1, and enhanced the expression of HSP70 [79]. Senoo, Sasaki [80] found that GGA may be used to treat liver fibrosis because it reduced fibrogenic activity, caused apoptosis in human HSCs in culture, and inhibited hepatic fibrosis in mice.

#### Geldanamycin analog (AAG)

HSP70 expression is increased in mouse microglia and neurons after 17-N-allylamino-17-demethoxygeldanamycin (17-AAG) treatment [81]. Zhang, Zhang [82] found that treatment with the HSP90 inhibitor 17-AAG could activate caspase-8 and caspase-9 and prevent NF-kB activation, leading to a significant increase in HSCs apoptosis. Additionally, when treated with 17-AAG, it reduced α-SMA expression and inhibited collagen synthesis induced by lipopolysaccharide and TGF-β_1_, suggesting that HSP90 is also involved in HSCs.

#### Cyclopentenone prostaglandins (cyPGs)

Cyclopentenone prostaglandins (cyPGs) have a cellular induction effect on HSP 70. HSP 70 overexpression inhibits viral infectivity factor [83]. The physiological resolution of inflammation is mediated by cyPGs, through the initiation of a genuine heat shock response (HSR), which includes cyPG-dependent activation of the HSF1 and expression of HSP70 [84].

#### AR-12

AR-12, derived from celecoxib, is an orally bioavailable, small-molecule inhibitor of phosphoinositide-dependent kinase-1 (PDK1) with potential antineoplastic activity. AR-12 inhibits the phosphorylation of PDK-1 and thus inhibits the activation of the serine/threonine protein kinase Akt (protein kinase B or PKB) [85], tumor cell proliferation, and inducing tumor cell death [86]. AR-12 decreased HSP90 and GRP78 protein levels while increasing HSP70 expression [87].

#### Zofenopril

In the chronic administration of zofenopril, an angiotensin-converting enzyme inhibitor, significant upregulation in gene expression of HSP70 was detected [88].

#### Anti-malaria drugs

The anti-malaria drugs quinacrine and emetine inhibited HSR in cancer cells by inducing hsp70 expression [89].

#### Prostaglandin E1 and lithium

Co-administration of PGE1 and lithium significantly increased cytoprotective HSP70 and HO-1 protein levels in a rat model of cerebral ischemia [90]. El-Ashmawy, Al-Ashmawy [91] found that lithium chloride promotes liver fibrosis and stimulates Wnt/β-catenin signaling. This makes the treatment for liver fibrosis using this combination less effective.

#### Ethanol

In the brain and liver, ethanol affects intracellular levels of GSH, HSP70, and protein carbonyls. There was a significant decrease in GSH, an increase in HSP70, and protein carbonyls in the brain, striatum, and hippocampus after seven days of ethanol treatment. Ethanol stimulates a redox mechanism that induces HSP70 induction in the brain [92].

#### Bleomycin

Bleomycin is an antineoplastic antibiotic that successfully induces HSP70. Bleomycin analog was reported to induce HSP70 in a pheochromocytoma cell line as well as several T-cell and monocytic cell lines. Cellular toxicity is produced by increasing the concentrations of these compounds that promote HSP70 mRNA [93]. Bleomycin produces distinct organ fibrogenesis as a net impact, even though its favorable effect on HSP70 is regarded [94].

**Fig. 1:**
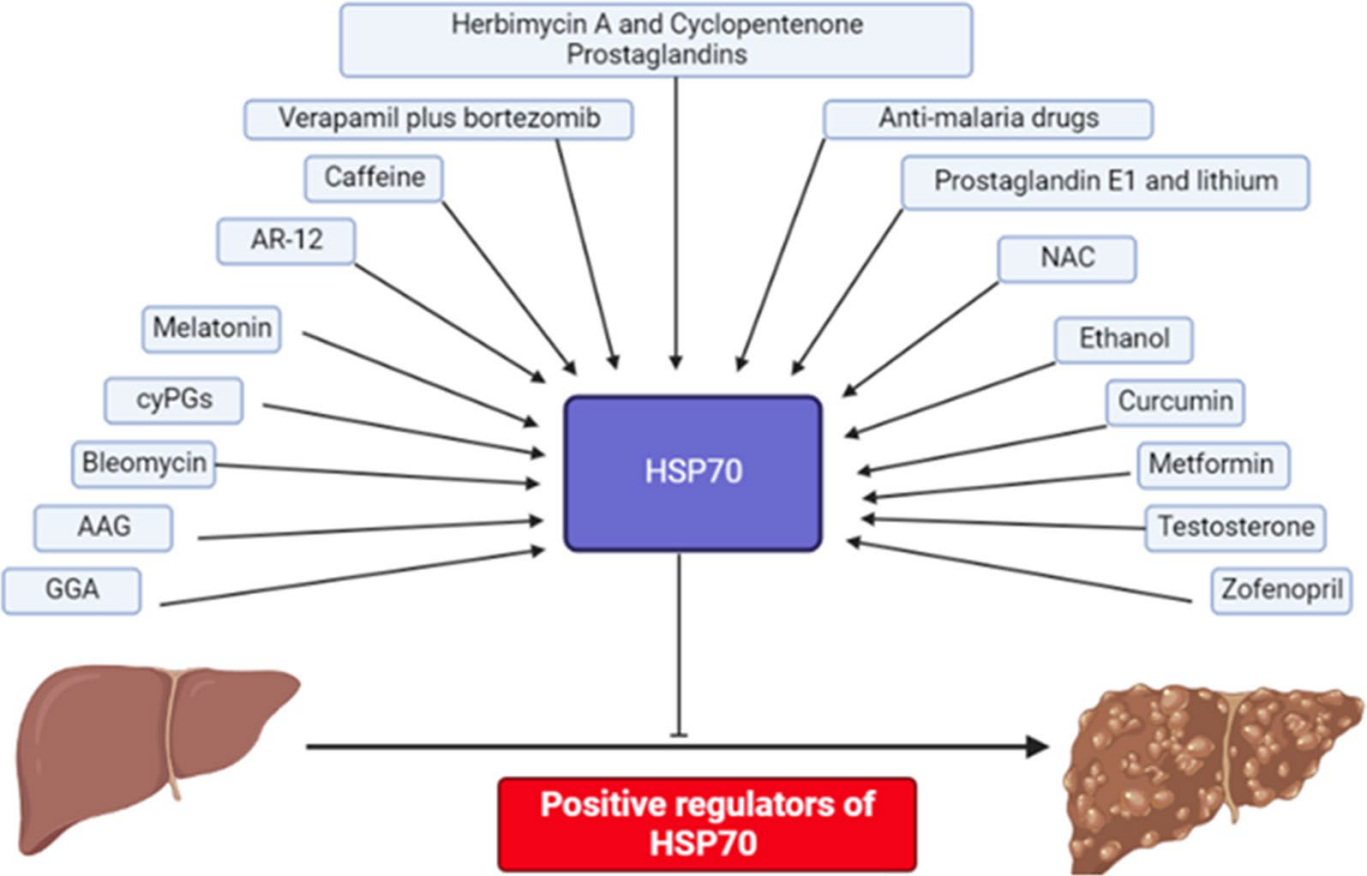
Positive regulators of HSP70

The following figure, Fig. 1, summarizes the data regarding HSP70 positive regulators.

### Negative regulators of HSP47

#### Aspirin

Aspirin appeared to have a protective effect against the renal damage caused by stress through its inhibitory effect on HSP60 and HSP47-mediated pathways [95, 96]. Aspirin significantly reduced liver inflammation and fibrosis through inhibition of HSC activation and proliferation, which led to a decrease in inflammatory markers such as IL-6, TNF-α, TLR4, MyD88, and NF-kB in those cells [97]. Sun, Liu [98] found that in the liver fibrosis model of rats, aspirin improved the degenerative abnormalities in the liver tissues. In a cross-sectional analysis, aspirin use was associated with significantly lower indicators of liver fibrosis in US individuals with suspected chronic liver diseases [99].

#### N-acetylcysteine

N-acetylcysteine had therapeutic value on liver fibrosis in the rat model [100]. Pereira-Filho, Ferreira [73] found that through histological investigation and collagen quantification, the cirrhotic group treated with NAC demonstrated decreased degree of fibrosis, less cellular membrane deterioration, and less expression of inducible nitric oxide synthase.

#### Pirfenidone

Pirfenidone (5-methyl-1-phenyl-2-(1H)-pyridone) is an anti-inflammatory anti-fibrotic drug that blocks the process of fibrosis in idiopathic pulmonary fibrosis patients and animal models. The antifibrotic effect of pirfenidone action is mediated through the inhibition of TGF-β_1_ and HSP47 expression. Xi, Li [101] found that by inhibiting Glrx, pirfenidone therapy prevents HSC activation and liver fibrosis. In HSCs, pirfenidone promotes Glrx expression in a STAT5-dependent way. Flores-Contreras, Sandoval-Rodríguez [102] found that two years of pirfenidone therapy reduces fibrosis in patients with chronic hepatitis C.

#### Vitamin C

Vitamin C can reduce cadmium toxicity by inhibiting changes in bioaccumulation, and hematological parameters such as calcium, magnesium, glucose, alkaline phosphatase (ALP), ALT, AST, total protein, lactate dehydrogenase (LDH), cholesterol, and lysozyme (LZM), and HSP-related genes (Hsp70, Hsp90, Hsp47, and Hsp60). Vitamin C has the potential to reduce heavy metal damage while also improving immunity [103]. Zhao and Li [104] cross-sectional study showed that there is an association of serum vitamin C with significant fibrosis in men and overweight or obese patients with NAFLD.

#### Tetrandrine

The in vivo comparing studies on BDL rats revealed a marked decrease in the quantification of Hsp47, collagen 1, α-SMA, and Pcol1A1 in precision-cut liver slices from fibrotic rat livers post-tetrandrine treatments [105, 106]. Hsu, Chiu [107] found that tetrandrine dramatically decreased the amount of hepatic collagen in dimethylnitrosamine-induced fibrosis in rats. Tetrandrine treatment reduced the number of NF-kB and α-SMA positive cells in the fibrotic livers. Tetrandrine therapy reduced the mRNA expression of intercellular adhesion molecule 1, α-SMA, and TGF-β_1_ and decreased plasma AST and ALT activity levels. Yin, Lian [108] found that tetrandrine encourages the apoptosis of activated HSCs.

#### Angiotensin II receptor 1 antagonist

Angiotensin II receptor 1 (AT1) antagonist candesartan maintained antifibrotic effects more effectively than ramipril in a randomized controlled prospective study involving 64 patients with chronic hepatitis C and liver fibrosis and may represent a secure and efficient therapeutic approach for liver fibrosis in patients with chronic liver diseases [109].

#### Dehydroepiandrosterone (DHEA)

Dehydroepiandrosterone treatment reduces the levels of androgen receptor (AR), procollagen 1 and 3, and HSP47 in the skin of postmenopausal women. DHEA significantly increased AR levels in the epidermis. A significant increase in the expression of types 1 and 3 procollagens, as well as HSP47, a procollagen chaperone protein, was observed in the dermis [110]. The potent stimulatory effect of topical DHEA on the number and size of dermal fibroblasts as well as the expression of procollagen types 1 and 3 suggests that topical DHEA may be a useful anti-aging agent in the skin [111]. Low levels of circulating DHEA-S are related to more severe NAFLD, which is denoted by the presence of NASH with the advanced fibrosis stage [112].

#### SB203580

SB203580 (4-(4-fluorophenyl)-2-(4-methyl sulfinyl phenyl)-5-(4-pyridyl)-imidazole) is a stress kinase inhibitor. It inhibits p38 MAPK through the block of MAPKAPK-2 activation and HSP phosphorylation [113]. Stress kinase inhibitor SB203580 downregulated collagen XVIII, CBP2/Hsp47, and VEGF expression induced by hypoxia [114]. Gao, Sun [115] found that SB203580 reduced the degree of liver fibrosis.

#### LY2109761

TGF-β has a significant role in metastasis and angiogenesis of cancer cells which could be inhibited by a small molecule inhibitor, LY2109761 [116]. LY2109761 has an inhibitory impact on the expression of HSP47 in rat precision-cut liver slices which is increased by the prolongation of incubation periods [117].

#### Imatinib

Imatinib treatment in hypertensive rats reduced PDGF-C, VEGF, HSP47, and HSP47 expression in the pulmonary veins, as well as the expression of α-SMA-positive cell proliferation [118]. When compared to traditional imatinib, HSC-targeted imatinib therapy exhibits remarkable anti-fibrotic benefits with less cytotoxicity [119]. Yoshiji, Noguchi [120] in vitro study showed that imatinib significantly reduced the proliferative and migratory effects of PDGF-BB as well as the mRNA levels of α-SMA and alpha2-(I)-procollagen in activated HSC in a dose-dependent manner. Additionally, imatinib dramatically reduced the phosphorylation of Akt, MEK1/2, and PDGFR-beta that PDGF-BB-induced in activated HSC. Unlike sorafenib, imatinib appears to merely diminish early liver fibrogenesis while not preventing long-term progression [121]. Prophylactic imatinib significantly reduced fibrosis in the first three weeks following bile duct ligation (BDL) in rats [122].

#### Sorafenib

Sorafenib was also found to significantly reduce the expression of fibrosis markers like α-SMA, Pcol1A1, and Hsp47 [123, 124]. Yuan, Wei [125] observed that HSC ferroptosis and ECM decrease caused by sorafenib were prevented by Fer-1 and DFO. Chen and Ji [126] observed that hepatic structure and fibrotic progression were improved and the expression of genes linked to fibrosis was dramatically decreased by sorafenib. Sorafenib prevented collagen I and α-SMA accumulation and reversed protein lysine crotonylation brought on by CCl4 injection. Pesce, Ciurleo [127] observed no additive or synergistic antifibrogenic effects for imatinib and sorafenib.

#### Sunitinib

Sunitinib is an indolin-2-one structural analog that is taken orally and inhibits various RTKs including VEGFR1/2/3, PDGFR, FGFR, and c-Kit [128]. Sunitinib demonstrated potent anti-tumor and anti-angiogenesis effects in a variety of cancer types in clinical trials. Sunitinib has been proven in liver fibrosis models to reduce inflammatory infiltration and the expression of fibrosis markers in liver fibrosis like HSP47 [129]. Sunitinib reduced collagen synthesis in HSCs, reduced HSC contraction, and reduced cell migration. Sunitinib inhibited the angiogenic potential of endothelial cells. Sunitinib was also found to decrease the number of VCAM-1 and ICAM-1 positive hepatic vasculature, as well as portal vein pressure, in cirrhotic rats [129]. Accordingly, Sunitinib dramatically lowers hepatic vascular density, inflammatory infiltrate, the abundance of α-SMA, LX-2 viability, collagen expression, and portal pressure in cirrhotic rats, which in turn reduces fibrosis and portal pressure as well as inflammatory infiltration.[130].

#### Meloxicam

Meloxicam reduced the expression of both HSP47 protein and type IV collagen mRNA which explains the improvement in mice unilateral ureteral obstruction (UUO)-induced renal interstitial fibrosis [130]. Meloxicam, a selective COX-2 inhibitor, inhibits BDL-induced hepatic fibrosis, which is accompanied by decreased hepatic TGF-β_1_expression and cyclooxygenase activity [131].

#### Emodin

Emodin is obtained mainly from Polygonaceae and is the active ingredient in *Reynoutria japonica Houtt*., and *Rheum palmatum L*. Emodin has antibacterial, antiviral, antitumor, and liver-protective properties [132, 133]. Emodin can minimize pulmonary edema and fibrosis, decrease collagen formation, and inhibit myofibroblast and inflammatory cell infiltration in the treatment of idiopathic pulmonary fibrosis (IPF). After bleomycin therapy, emodin lowered the levels of TNF-α, IL-6, TGF-β_1_, and HSP-47 in lung tissue [134]. Emodin can lessen the severity of liver fibrosis by decreasing the infiltration of Gr1hi monocytes and drastically reducing the production of granulin (GRN), monocyte chemoattractant protein 1 (MCP-1), TNF-α, TGF-β_1_, and chemokine ligand 7 (CCL7) in the liver [135].

#### Nintedanib and pirfenidone

Both medications influence critical regulatory levels in collagen synthesis and processing. Both drugs inhibited collagen I fibril formation and reduced and altered the appearance of collagen fibril bundles, indicating that both drugs have a completely new mechanism of action [136]. Nintedanib effectively inhibited profibrotic gene expression and collagen secretion. The regulation of the collagen chaperone FKBP10 was consistently down-regulated by nintedanib in IPF fibroblasts but not in donor fibroblasts. Pirfenidone reduced FKBP10 transcript while increasing FKBP10 protein levels in donor fibroblasts, despite not affecting FKBP10 expression in IPF fibroblasts. Nintedanib had a greater negative effect on HSP47 transcription in IPF fibroblasts than in donor fibroblasts [137, 138]. The results of the trial show that nintedanib has an antifibrotic and anti-inflammatory effect outside of the lungs. It helped lower hepatic damage, inflammation, and fibrosis in both the preventative and therapeutic treatment schedules [139]**.**

#### Caveolin-1

Caveolin-1 (Cav-1) is a supporting protein that is essential for the formation of caveolae plasma membranes in most cell types. Cav-1 expression is found in most normal organs, but it is reduced when tissue is isolated or grown in culture [140]. Cav-1, due to its decreased expression in fibroblasts and monocytes, is essential in fibrosis in various tissues. Cav-1 reduced the levels of collagen I, HSP47, fibronectin, and CTGF, as well as the activation of the non-receptor tyrosine kinases Pyk2 and Src and the activation of eNOS [141].

#### ND-L02-s0201

NDT-05–0038 is a nuclease-resistant, synthetic, double-stranded small interfering ribonucleic acid (siRNA) developed to reversibly suppress the production of HSP47 by targeting the homologous region across humans, rats, and mice [142]. ND-L02-s0201 revealed strong antifibrotic effects and improved lung function in two robust chronic rodent models of pulmonary fibrosis, supporting its use in people with idiopathic pulmonary fibrosis [142].

#### Corticosteroids

Total ECM and collagen deposition were inhibited by corticosteroids via the glucocorticoid receptor and Hsp47 mRNA expression. Budesonide inhibited the mRNA expression of Hsp47 [143, 144]. Shimizu, Shimizu [145] found that an increase in corticosteroid dosage may raise the chance of developing NAFLD and liver fibrosis.

#### Valproic acid

Valproic acid is an anticonvulsant and mood stabilizer medication. It is widely used in the adult population to treat convulsions, migraines, and bipolar disorders. In terms of the antifibrotic investigated, sunitinib and valproic acid might lower HSP47 and PCOL1A1 gene levels [146]. AST, ALT, ALP, and GGT serum enzyme activity all increased significantly after taking valproic acid. Additionally, it markedly decreased lowered GSH content while considerably increasing MDA and NO. Valproic acid delivery simultaneously caused a significant rise in hydroxyproline, TNF-α production, and NF-kB expression, increasing the risk of liver fibrosis development [147].

**Fig. 2:**
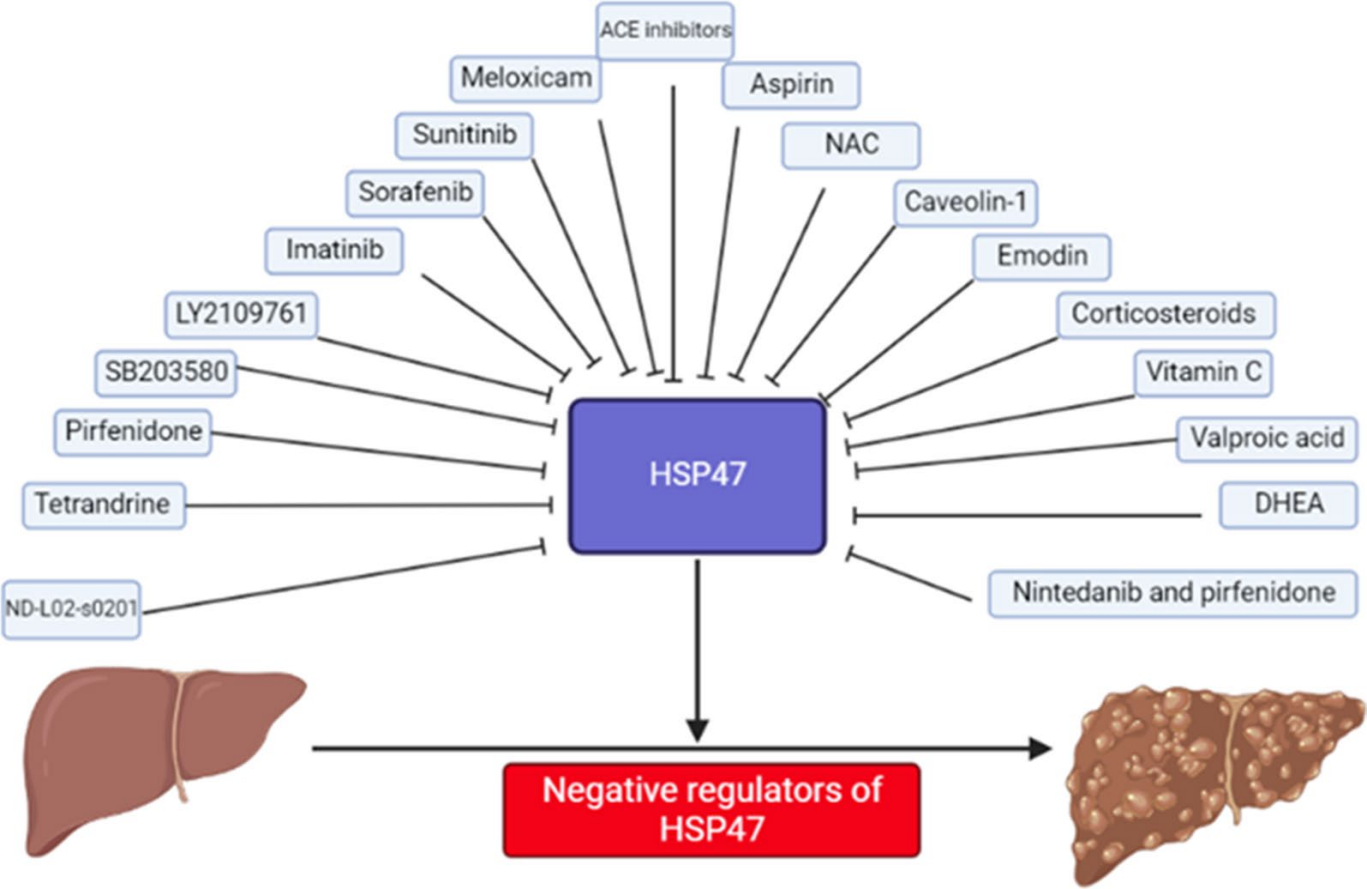
Negative regulators of HSP47

The following figure, Fig. 2, summarizes the data regarding HSP47 negative regulators.

**Fig. 3:**
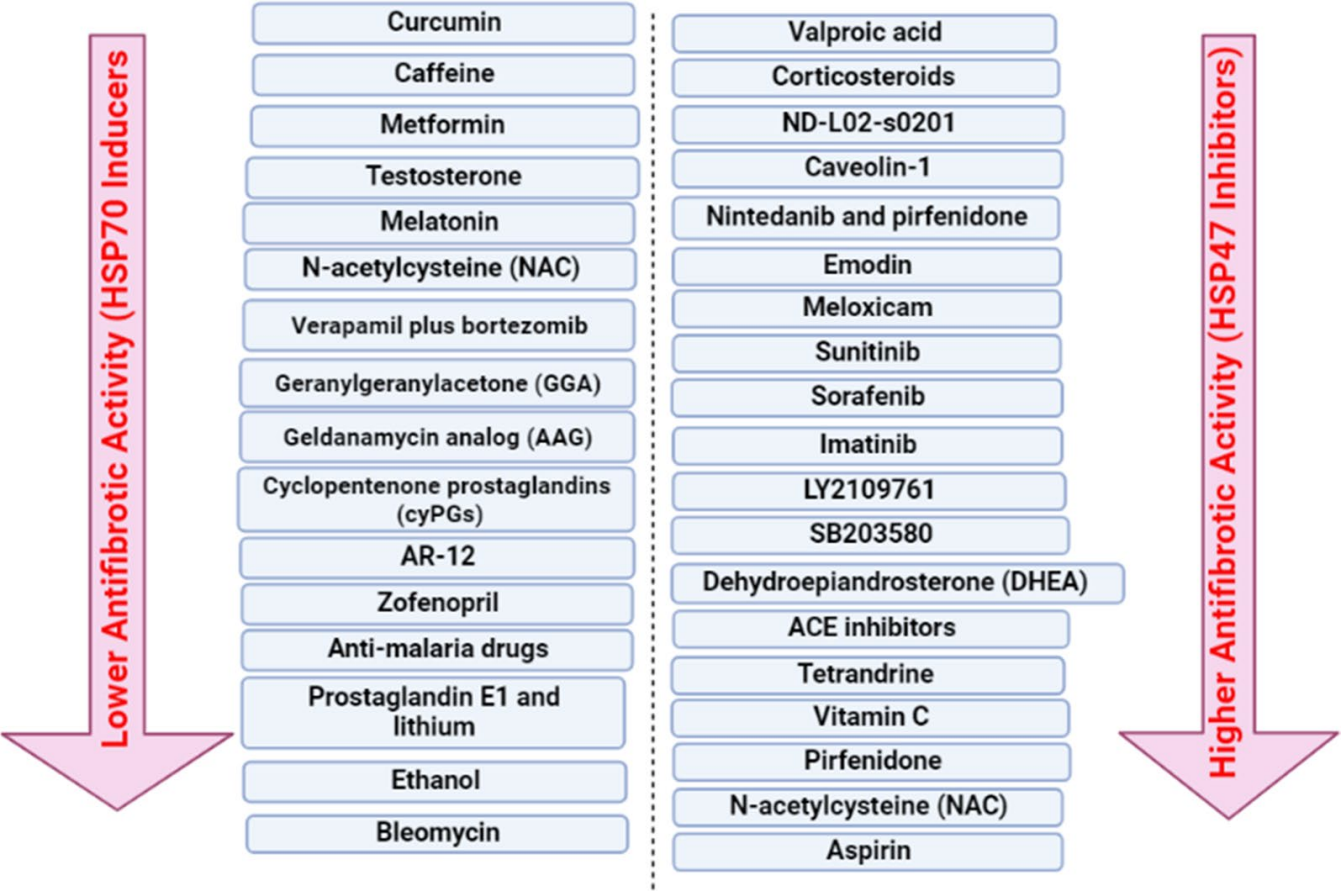
HSP47 and HSP70 targeted drugs. These drugs ranked according to applicability in previous studies in both experimental and clinical efficacy, then that of only experimental effect and the latter types that have a conflict regarding efficacy whatever the potency against HSPs

The following figure, Fig. 3, summarizes the data arranging drugs that may be most promising to treat hepatic fibrogenesis. The highest priority that approved both experimental and clinical efficacy, then that of only experimental effect and the latter types that have a conflict regarding efficacy whatever the potency against HSPs.

## Conclusion

As HSP70 and HSP47 are potential targets for the control of liver fibrosis due to their role in the regulation of HSCs activation, collagen synthesis, and fibrogenesis, drugs that inhibit HSP47 or induce HSP70 can be tested for their effectiveness against liver fibrosis, especially for comorbidities.

## Data Availability

Not applicable.
